# Short and long term central peripherally inserted catheters in hematological malignancy patients: clinical outcomes and safety. Single centre experience

**DOI:** 10.3332/ecancer.2025.2044

**Published:** 2025-11-20

**Authors:** Carlos Gómez Calcetero, Juanita Granados Diaz, Adriana Aya Porto, Andres Forero Romero, Maria López Mora, Paola Omaña Orduz, Jorge Daza Buitrago, Maira Murcia Linares, Viriginia Abello Polo

**Affiliations:** 1 Clinical Functional Unit of Leukaemia, Lymphoma and Myeloma, Luis Carlos Sarmiento Angulo Cancer Treatment and Research Centre (CTIC), Bogotá 110141, Colombia; 2 Research Group GIGA, CTIC/Universidad El Bosque, Bogotá 110141, Colombia; 3 Research and Education Institute, Luis Carlos Sarmiento Angulo Cancer Treatment and Research Centre (CTIC), Bogotá 110141, Colombia; 4 Clinical Functional Unit of Intensive Care, Luis Carlos Sarmiento Angulo Cancer Treatment and Research Centre (CTIC), Bogotá 110141, Colombia; 5 Infectious Diseases Service, Luis Carlos Sarmiento Angulo Cancer Treatment and Research Centre (CTIC), Bogotá 110141, Colombia

**Keywords:** hematologic neoplasms, catheter-related infections, vascular access devices, thrombosis

## Abstract

**Objective:**

To characterise the population and describe the complications in patients with hematologic malignancies who underwent implantation of a peripherally inserted central catheter (PICC) or midline catheter (MC) for the administration of chemotherapy or oncologic support.

**Methods:**

The retrospective descriptive study included patients with hematologic malignancies who underwent PICC or MC implantation between July 2022 and 2024. All patients are part of the institution-based prospective observational Evidence – Verification – Analysis study. Variables related to device type were obtained from the vascular access and infection control group databases. Frequencies and percentages were used for categorical variables, and medians and interquartile ranges were used for numerical variables. Event rates per 1,000 days were calculated for complications.

**Results:**

156 patients with 249 events were included. All devices were high-flow and bi-lumen, the most common indication was chemotherapy administration, and the most common site was brachial. The median days in use for lymphoma and acute leukaemia were 67.5 and 24 days, respectively. The rates of catheter-associated thrombosis and catheter-associated bloodstream infection were 3.2% and 3.7%, respectively. The rates of catheter-related venous thrombosis per 1,000 catheter days and catheter-related bloodstream infection per 1,000 catheter days were 0.31 and 0.83, respectively.

**Conclusion:**

A multidisciplinary approach, thorough initial venous assessment, a strong support network, adherence to care protocols and continuous patient education have helped to reduce variability in care and lower complication rates at our institution.

## Background

Peripherally inserted central catheters (PICCs) are widely used in patients requiring medium- to long-term intravenous therapy with osmolarity >600 mOsm and pH <5 or >9 for hematologic malignancies; they are used to administer chemotherapy and oncologic support, including blood transfusions, antibiotic therapies, total parenteral nutrition and to obtain blood samples for analysis [1–4].

The advantages of PICCs over other central vascular access devices include bedside insertion without the need for intravenous anesthesia or sedation, reduced risk of pneumothorax and bleeding during catheter insertion and reduced risk of bleeding in patients with thrombocytopenia [5, 6].

Midline catheters (MCs) are also used for patients requiring short to mild-term (7–28 days) intravenous chemotherapy and supportive therapy. Their advantages include administration of chemotherapeutic agents and iodinated contrast media with osmolarity <600 mOsm and pH between 5 and 9, and supportive therapy with a lower rate of complications, including mechanical and bacterial phlebitis and a lower risk of local infection [7].

However, there is a plethora of complications associated with PICCs and MCs. These complications include catheter-related bloodstream infections (CRBIs) [8], catheter-related venous thrombosis (CRVT) [9] and mechanical issues such as occlusion, rupture, malposition or accidental withdrawal [10].

The Luis Carlos Sarmiento Angulo Cancer Treatment and Research Centre (CTIC) and its Clinical Functional Unit for leukaemia, lymphoma and myeloma (CFU-LLM) initiated operations in 2022. Following a comprehensive review of vascular access options for patients during a multidisciplinary meeting, the CTIC vascular access team (VAT) and the CFU-LLM reached a consensus that PICCs or MCs were the optimal choice for our patients. It was determined that all vascular access devices utilised should be high-flow and bilumen.

Subsequent to the initial diagnosis, each patient undergoes evaluation by a multidisciplinary committee, in accordance with the established guidelines of the VAT, to determine the optimal vascular access method for each individual case. The evaluation encompasses a comprehensive assessment of clinical parameters, including diagnosis, age and chemotherapy regimen, as well as social, educational and psychological factors. The present study aims to describe our experience using PICCs or modified central catheters (MCs) for chemotherapy and hematological care and assess the potential benefits and risks associated with the use of these central vascular access devices in this specific patient population.

## Methods

### Study design

The present descriptive, retrospective observational study aims to evaluate the rate of catheter-related complications and dwell time of each device for patients diagnosed with hematological malignancies who required PICC or MC placement at CTIC from August 2022 to 2024. Each patient is included in the global institution's prospective observational study evidence – verification – analysis (EVA), which was approved under ACTA CEI-114 to ensure compliance with ethical guidelines and to obtain informed consent. The CTIC Scientific Committee has formally endorsed a sub-analysis of this particular patient population from EVA.

### Selection and description of participants

Participants were required to be over the age of 18 and were selected from cases enrolled in the EVA after signing the informed consent form. The present analysis included patients treated at the CFU-LLM who were diagnosed with hematological diseases, including lymphomas, myeloma, acute leukaemias and chronic leukaemias, who required intravenous chemotherapy and support treatment through PICCs or MCs between August 2022 and 2024. Cases with incomplete medical records were excluded from the study.

**Figure 1.**
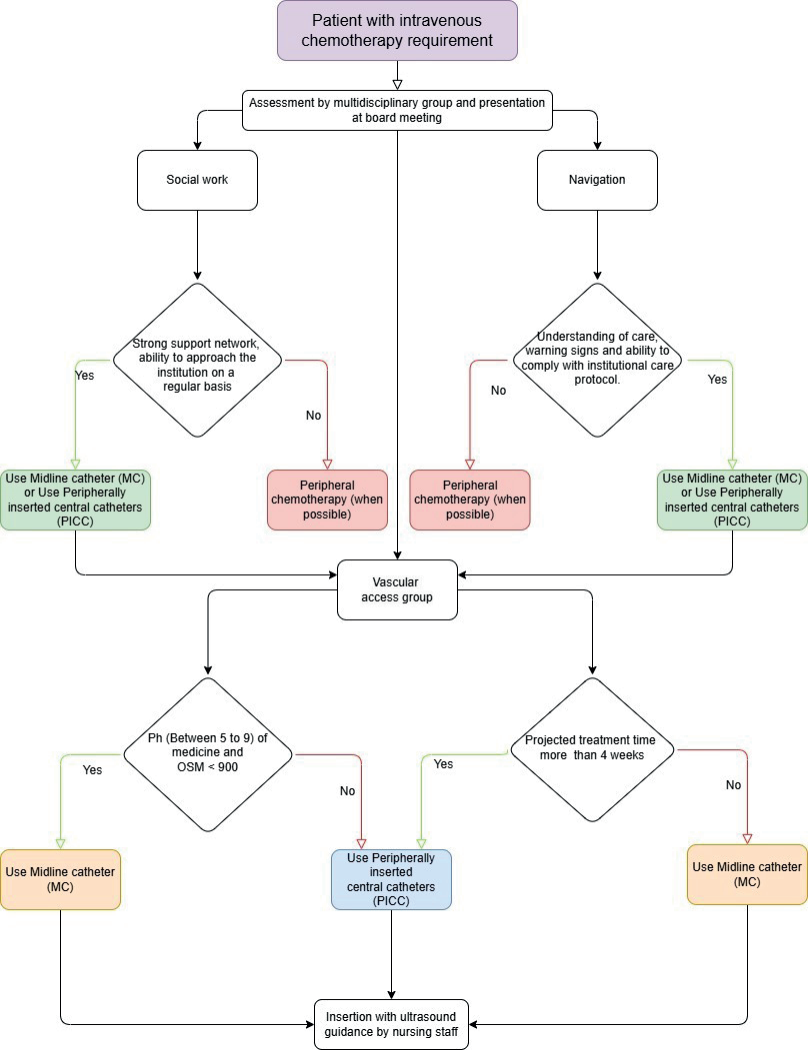
Decision algorithm for vascular device implantation.

The institutional model of patient- and family-centred care empowers patients to engage in navigation, a process that aims to remove barriers for vulnerable and resource-limited populations [11, 12]. At the time of diagnosis, the treating hematologist typically initiates the process of determining the most suitable vascular access for each patient. The nurse navigator plays a pivotal role in orchestrating the efforts of the multidisciplinary team, ensuring comprehensive education on device care, identifying potential red flags necessitating consultation with the facility and devising a personalised follow-up plan for each patient. The decision to implant a central access device is made by the multidisciplinary committee, which convenes weekly to discuss all new diagnoses and reach a consensus opinion. The social work department is responsible for defining the patient's support network and the economic resources available to attend the centre with the frequency indicated according to the institutional vascular access care protocol. The management of catheter-related complications is carried out in accordance with the established institutional protocols. Finally, the VAT evaluates the patient's venous capital and determines the most appropriate device according to the algorithm presented in Figure 1.

### Outcome definitions

CRBI is defined as bacteremia occurring at least 48 hours after the insertion of a PICC or MC, with the isolation of a microorganism not considered a contaminant and not related to infections at another site. The presence of the same microorganism in both the central blood culture and the peripheral blood culture is required for a positive diagnosis. Additionally, a time lag of more than 2 hours between positive blood cultures from the central and peripheral sites is considered diagnostic of a CRBI, provided that the central blood cultures test positive earlier than the peripheral ones [13, 14].

Non-catheter-related bloodstream infection (NCRBI) is characterised by the presence of bacteremia that does not fulfill all the criteria for CRBI [15].

CRVT is defined by the presence of thrombus identified by ultrasonography in patients exhibiting signs of acute thrombosis [16]. The occurrence of deep vein thrombosis or superficial vein thrombosis was regarded as an event.

Mechanical complications are defined as occlusion, rupture, malposition or accidental withdrawal [2, 10].

Catheter failure is defined as a range of complications that result in the premature removal of a PICC or MC.

The dwell time of a catheter is defined as the total time in days that the device remains inserted and operational, commencing from the time of successful insertion and ending at the time of removal.

### Data collection and measurements

The data were systematically gathered through a centralised information platform designed for dynamic reference capture, allowing for logical independence and minimal redundancy via data columns. The primary objective of the study was to evaluate complications associated with central venous access, while secondary objectives included analysing demographic and clinical outcomes.

The data sources comprised electronic medical records and laboratory reports, ensuring the collection of high-quality and structured information. To ensure the reliability of the data, each variable was meticulously defined and measured, with particular attention devoted to the accuracy of the information captured.

Furthermore, artificial intelligence tools, namely ChatGPT (OpenAI, San Francisco, USA) and Gemini (Google DeepMind, Mountain View, USA), were utilised to evaluate and enhance the process of data collection. This ensured clarity, consistency and compliance with best practices in medical documentation.

### Statistical analysis

Statistical analyses were conducted using Python version 3.11.4. Quantitative variables were summarised using medians and interquartile ranges (IQRs), while categorical variables were presented as frequencies and percentages. The incidence of complications was determined by calculating the number of complications per 1,000 days of catheter use.

To assess the statistical significance of the findings, appropriate statistical tests were employed. The manipulation of data and the execution of statistical computations were carried out using the Python programming language, with the specific libraries Pandas and NumPy being utilised for the analysis and statistical computation.

### Ethical considerations

The present study was conducted in adherence to the principles established in the Declaration of Hensinki. This study is part of the prospective observational study EVA, which was submitted to the institutional research committee, which approved the performance of a sub-analysis for this study.

## Results

### Demographics and clinical characteristics

The study population comprised 156 patients diagnosed with hematologic malignancies and 249 vascular access devices, including PICCs and MCs. The median age of the entire cohort was 60 years (IQR 45–70). The primary therapeutic approaches for lymphoma included R-CHOP-such as regimens, ABVD and infusion protocols. In patients diagnosed with acute leukaemia, the utilisation of PICC was exclusive to remission induction protocols. Table 1 delineates the demographics and clinical characteristics of the patients included in the study.

### Characteristics of vascular access devices

**Figure 2.**
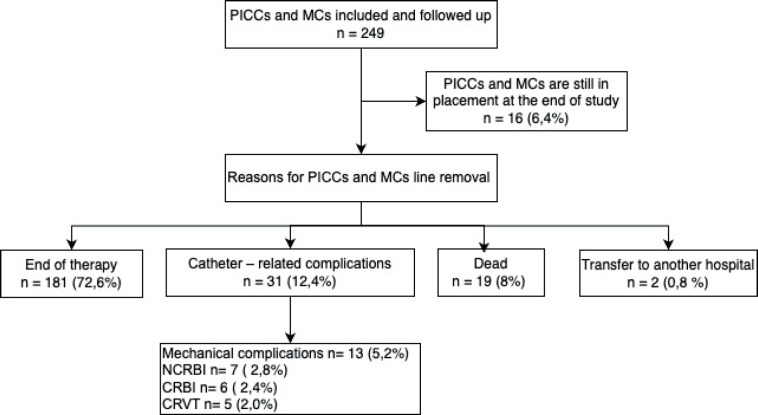
Flowchart of reasons for PICCs or MCs line removal. PICCs: Peripherally inserted central catheters, MCs: Midline catheters, CRBI: Catheter-related bloodstream infection, NCRBI: Non-catheter-related bloodstream infection, CRVT: Catheter-related venous thrombosis.

With respect to the characteristics of vascular access devices, peripherally inserted PICCs exhibited a higher prevalence compared to MCs. The basilic vein emerged as the predominant placement location, followed by the brachial vein. The predominant indication for both PICCs and MCs placements was chemotherapy (95.1%), with the primary cause for removal being the completion of therapy (72.6%), followed by catheter failure (13.2%) and death due to causes unrelated to catheter complications (8%). Table 2 delineates the types and characteristics of vascular access devices categorised by pathology, and Figure 2 illustrates the reasons for the removal of PICCs and MCs.

### Catheter related complications

With respect to catheter-related complications, mechanical complications were the most prevalent, followed by NCRBIs and CRBIs. The median PICC and MC dwell time in days for the entire cohort was 43 (IQR 17–98) days. The median PICC and MC dwell time in days for the group that presented with catheter failure was 27.5 (IQR 14.75–59) versus 46 (IQR 17–104) in the group that did not present.

The proportion of CRBI, NCRBI, CRTV and mechanical complications was as follows: 3.7%, 4.4%, 3.2% and 8.3%, respectively. The rate of CRTV and CRBI per 1,000 catheter days was 0.31 and 0.83, respectively. It is noteworthy that no fatalities occurred due to catheter-related complications, and no instances of pulmonary embolism were observed that were attributable to CRTV.

## Discussion

The findings of our study demonstrate that PICCs and MCs are both safe and effective vascular devices for the delivery of medium- and long-term hematological treatments. They also serve as an excellent tool for the intensive supportive care required in a hematology ward. However, evidence on the use of PICCs and MCs in hematology patients is scarce and more prospective studies to evaluate the clinically relevant outcomes of these devices in this specific population are needed.

The best available information for using PICCs in chemotherapy treatment is provided by The Cancer and Venous Access trial, which included more than 1,000 patients with solid tumours or hematologic neoplasms who underwent placement of tunneled catheters, PICCs or totally implanted PORTs. The primary outcome measured was the overall complication rate, which was a composite of suspected or confirmed infection or mechanical failure [17].

After a 1-year follow-up, the results showed that complications were less common in patients with PORTs compared to tunneled catheters (29% versus 43%; OR 0.54 (95% CI, 0.37–0.77)) and patients who received PORTs had better results compared to those who received PICCs (32% versus 47%; OR, 0.52 (95% CI, 0.33–0.83)). The infection rate was higher with PORTs than PICCs (12% versus 8%) and the rate of CRBIs for PORTs compared to tunneled catheters was lower for PORTs (0.02 versus and 0.06 per catheter week). Additionally, removal was more frequent with PICCs than PORTs (38% versus 24%) [17].

Although this study concluded that PORTs are more effective and safer than both tunneled catheters and PICCs, it is important to highlight that the population with hematological neoplasms is not sufficiently represented in this series, which only includes 10% of patients with these pathologies [17].

Derudas et al [18, 19] published an Italian single-centre experience regarding the use of PICCs for the inpatient and outpatient management of patients diagnosed with acute lymphoblastic leukaemia, Hodgkin's and non-Hodgkin's lymphomas. Their results underlined that these devices are a safe and effective tool in the management of these diseases [18, 19]. Notably, this is the first report detailing the exclusive use of PICCs and MCs in patients with hematological malignancies.

In Latin America, there have been some published experiences from Brazil regarding the use of PICCs in oncological patients with remarkable results for antineoplastic therapy, diagnostic tests, curative measures, analgesia, nutritional support and sedation. Complications necessitating catheter removal occurred in fewer than 30% of cases [3]. However, it is noteworthy that only 11% of the patients represented in this study had hematological neoplasms. These results highlight the importance of evaluating the exclusive use of these devices in this specific population.

The Colombian experience regarding the use of PICCs and MC is limited, with only one report published by Vélez et al [20] outlining the use of PICC for indications other than chemotherapy treatment, specifically in non-oncological population in a fourth level Colombian hospital. To the best of our knowledge, our study represents the first Colombian description of the safety and efficacy of PICCs and MCs in patients exclusively with hematological malignancies [20].

Regarding catheter failure, in our experience, 72.6% of the devices inserted at diagnosis were retired upon completion of the hematological treatment. Similar outcomes were observed in the studies by Derudas et al [18, 19] where the finalisation of the treatment was also the main reason to remove the vascular devices (61% for ALL, 86.1% for HL and 76.5% for NHL) [18, 19]. Only 13.2% required early withdrawal due to failure.

The incidence of mechanical complications and both CRBI and CRVT was less frequent compared to the reported rates in the literature. Table 3 compares our findings with global data.

The decision to implant central access is made after consideration by the clinical team, the VAT, social work and navigation, all carrying equal weight. It is crucial not only to assess the anatomical viability of access but also to ensure that the patient has a robust support network to ensure proper device care. The education provided by the nurse navigator is paramount for patients and their families to comprehend the necessity of the device, the significance of maintenance and to recognise warning signs warranting consultation with the medical facility. Institutional commitment is also essential in device management, with dedicated scheduling for PICCs and MC maintenance and appropriately trained staff being vital for achieving optimal outcomes [22].

In our model, the multidisciplinary approach, which helps select the best vascular access option for each case, patient education, adherence to strict protocols for device placement and maintenance, may account for our excellent results [23].

This is a retrospective, single-centre, monographic study in a specific local patient population that aims to fill the local knowledge gap on the use of this type of vascular access in hematological cancer patients. Future prospective analytical and inferential studies are needed to validate the safety and efficacy of the use of PICCs and MCs in this patient subgroup and to assess the impact on hospital stay, overall survival, cost-effectiveness and quality of life.

## Conclusion

PICCs and MCs could be used effectively and safely in patients with hematological malignancies for the administration of chemotherapy and supportive treatment, offering some advantages such as bedside insertion facilities, lower risk of complications and reduced bleeding risk in patients with thrombocytopenia. However, evidence assessing catheter selection for medium to long-term use and describing complication rates, exclusively in patients with hematological malignancies, remains limited.

## List of abbreviations

CFU-LLM, Clinical Functional Unit for leukaemia, lymphoma and myeloma; CRBI, Catheter-related bloodstream infection; CRVT, Catheter-related venous thrombosis; EVA, Evidence – verification – analysis; MCs, Midline catheters; NCRBI, Non-catheter-related bloodstream infection; PICCs, Peripherally inserted central catheters; VAT, Vascular access team.

## Conflicts of interest

The authors declare no conflicts of interest.

## Funding

This research did not receive any specific grant funding agencies in the public, commercial or not-for-profit sectors.

## Author contributions

CFGC and AFFR made significant contributions to the conception and design of the work. AFFR, JGD and ACAP contributed to the acquisition of the study data. ACAP, CFGC and JGD contributed to the analysis and interpretation of the study data. All authors participated in the drafting and critical review of the manuscript. All authors read and approved of the final manuscript. All authors agreed to be accountable for all aspects of the work, ensuring that any questions related to its accuracy or integrity are appropriately investigated and resolved.

## Figures and Tables

**Table 1.** Demographics and clinical characteristics.

*n* = 156			
Characteristics	Lymphomas* (*n* = 101)	Acute leukemias^†^ (*n* = 42)	Chronic leukemia and multiple myeloma (*n* = 13)
Age, years (median, IQR)	62 (47–73)	50.5 (36.7–66.7)	68 (51–70)
Gender			
Male	58 (57.4)	23 (54.8)	8 (61.5)
Female	43 (42.6)	19 (45.2)	5 (38.5)
ECOG at diagnosis			
0–1	78 (77.1)	39 (92.8)	9 (69.2)
2–3	14 (13.9)	1 (2.4)	3 (23.1)
4	2 (2)	-	1 (7.7)
Not available	7 (7)	2 (4.8)	-
First line therapy			
R-CHOP like^‡^	75 (74.3)	-	-
Induction of remission in acute leukemia^§^	-	42 (100)	-
ABVD	11 (10.9)	-	-
Others^¶^	7 (7)	-	13 (100)
Infusional protocols in lymphoma^||^	4 (3.4)	-	-
Support therapy**	1 (1)	-	-
Second line therapy			
Platinum – based chemotherapy ^††^	1 (1)	-	-
Others^§§^	7 (7)	-	-

*Hodgkin and non-Hodgkin lymphomas. † Acute myeloid leukemia and acute lymphoblastic leukemia. ‡ Rituximab + CHOP21, R- Bendamustina, R- miniCHOP. § HyperCVAD, GRAALL, 7+3, FLAG –IDA, azacitidine + venetoclax. || DA – EPOCH. **Antibiotic therapy, total parenteral nutrition. †† ESHAP, GEM – P. ¶ Bedamustine + obinotuzumab, venetoclax + obinotuzumab, Bortezomib + Lenalidomide + Dexamethasone, Daratumumab + Bortezomib + Lenalidomide + Dexamethasone, Cyclophospamide + Bortezomib + Dexamethasone. §§ R - DHAP, Br – ESHAP, R – GemOx

**Table 2.** Characteristics of PICCs and MCs.

n = 249			
Characteristics	Lymphomas (*n* = 142)	Acute leukemia (*n* = 91)	Chronic leukemia and multiple myeloma (*n* = 16)
Type of central vascular access device			
PICC	124 (87.3)	61 (67)	10 (62.5)
MC	18 (12.7)	30 (33)	6 (37.5)
Placement location			
Basilic vein	96 (67.6)	48 (52.8)	11 (68.7)
Brachial vein	35 (24.6)	32 (35.1)	4 (25)
Axillary vein	7 (4.9)	2 (2.2)	1 (6.3)
Others	4 (2.8)	9 (9.8)	-
Indication for PICCs† or MCs‡ placement			
Chemotherapy	141 (99.3)	91 (100)	2 (12.5)
Supportive care	1 (0.7)	-	14 (87.5)
Median PICCs† and MCs‡ dwell time, days (median, IQR) Median PICCs† and MCs‡ dwell time, days (median, IQR)	67.5 (23.5–111.5)	24 (17–58.5)	17.5 (7–34.7)

†PICCs: Peripherally inserted central catheters, ‡ MCs: Midline catheters

**Table 3.** Incidence of catheter-related complications.

Catheter related complications	Our study	Reported in literature
Rate of CRBI × 1,000 catheter days	0.83	0.95–2.2 [3]
Rate of CRVT × 1,000 catheter days	0.31	0.51 [21]
Proportion of mechanicals complications*	8.3%	13.6% [21]

* Occlusion, rupture, malposition or accidental withdrawal

## References

[1] Gallieni M, Pittiruti M, Biffi R (2008). Vascular access in oncology patients. CA Cancer J Clin.

[2] Grau D, Clarivet B, Lotthé A (2017). Complications with peripherally inserted central catheters (PICCs) used in hospitalized patients and outpatients: a prospective cohort study. Antimicrob Resist Infect Control.

[3] Bonfim ALV, De Brito GA, Baptista AL (2023). Clinical study of complications of a peripherally inserted central catheter in cancer patients. Nurs Open.

[4] Campagna S, Gonella S, Berchialla P (2019). Can peripherally inserted central catheters be safely placed in patients with cancer receiving chemotherapy? a retrospective study of almost 400,000 catheter-days. Oncologist.

[5] Sapkota S, Sannur R, Naik R (2020). Analysis of peripherally inserted central catheter line in cancer patients: a single-center experience. South Asian J Cancer.

[6] Lu A, Hu M, Qi X (2024). A retrospective cohort study of implantable venous access port-related and peripherally inserted central catheter-related complications in patients with hematological malignancies in China. SAGE Open Med.

[7] Adams DZ, Little A, Vinsant C (2016). The midline catheter: a clinical review. J Emerg Med.

[8] Norris LB, Kablaoui F, Brilhart MK (2017). Systematic review of antimicrobial lock therapy for prevention of central-line-associated bloodstream infections in adult and pediatric cancer patients. Int J Antimicrob Agents.

[9] Yue J, Zhang Y, Xu F (2022). A clinical study of peripherically inserted central catheter-related venous thromboembolism in patients with hematological malignancies. Sci Rep.

[10] Greencorn DJ, Kuhle S, Ye L (2023). Risk factors for mechanical complications of peripherally inserted central catheters in children. Infect Control Hosp Epidemiol.

[11] Pautasso FF, Lobo TC, Flores CD (2020). Nurse navigator: development of a program for Brazil. Rev Lat Am Enfermagem.

[12] Lubejko B, Bellfield S, Kahn E (2017). Oncology nurse navigation: results of the 2016 role delineation study. Clin J Oncol Nurs.

[13] Osorio Lombana JP, Cely Andrade JL (2021). Documento de Actualización de Criterios Para la Notificación de Infecciones Asociadas a la Atención en Salud (IAAS) al sistema de vigilancia epidemiológica en Bogotá D. C.

[14] Centers for Disease Control and Prevention (2024). Bloodstream Infection Event (Central Line-Associated Bloodstream Infection and Non-central Line Associated Bloodstream Infection).

[15] Hoon Baang J, Inagaki K, Nagel J (2023). Inpatient Diagnosis and Treatment of Catheter-Related Bloodstream Infection.

[16] Linenberger ML (2006). Catheter-related thrombosis: risks, diagnosis, and management. J Nat Compreh Cancer Netw.

[17] Moss JG, Wu O, Bodenham AR (2021). Central venous access devices for the delivery of systemic anticancer therapy (CAVA): a randomised controlled trial. Lancet.

[18] Derudas D, Chiriu S, Romani C (2023). Peripherally inserted central catheter safety and efficacy in acute lymphoblastic leukemias: a 16-years monocentric experience. Blood.

[19] Derudas D, Simula MP, Massidda S (2022). PICC insertion and management in Hodgkin and NON-Hodgkin lymphomas: a 13-YEARS monocentric experience. Blood.

[20] Vélez P, Millán SL, Restrepo JG (2017). Experiencia en el uso de catéteres centrales de inserción periférica en una institución de cuarto nivel en Colombia, 2011–2014. Rev Colomb Hematol Oncol.

[21] Balsorano P, Virgili G, Villa G (2020). Peripherally inserted central catheter–related thrombosis rate in modern vascular access era—when insertion technique matters: a systematic review and meta-analysis. J Vascular Access.

[22] Curto-García N, García-Suárez J, Callejas Chavarria M (2016). A team-based multidisciplinary approach to managing peripherally inserted central catheter complications in high-risk haematological patients: a prospective study. Supp Care Cancer.

[23] Centro de Tratamiento e investigación sobre el Cáncer Luis Carlos Sarmiento Angulo (2022). Plan de Desarrollo 2023–2027.

